# Tree-ring history of Swiss needle cast impact on Douglas-fir growth in Western Oregon: correlations with climatic variables

**DOI:** 10.29328/journal.jpsp.1001065

**Published:** 2021-11-25

**Authors:** E Henry Lee, Peter A Beedlow, Ronald S Waschmann, Steve Cline, Michael Bollman, Charlotte Wickham, Nicholas Testa

**Affiliations:** 1 US Environmental Protection Agency, 200 SW 35^th^ Street, Corvallis, OR 97333, USA; 2 Department of Statistics, Oregon State University, Corvallis, OR 97331, USA; 3 Department of Transportation, Oregon State University Corvallis, OR 97330, USA

## Abstract

The fungal pathogen, *Nothophaeocryptopus gaeumannii*, occurs wherever Douglas-fir is found but disease damage is believed to be limited to the Coast Range and is of no concern outside the coastal fog zone (Shaw, et al., 2011). However, knowledge remains limited on the history and spatial distribution of Swiss Needle Cast (SNC) impacts in the Pacific Northwest (PNW). We reconstructed the history of SNC impacts on mature Douglas-fir trees based on tree ringwidth chronologies from the west slope of the Coast Range to the high Cascades of Oregon. Our findings show that SNC impacts on growth occur wherever Douglas-fir is found in western Oregon and is not limited to the coastal fog zone. The spatiotemporal patterns of growth impact from SNC disease were synchronous across the region, displayed periodicities of 25–30 years, strongly correlated with winter and summer temperatures and summer precipitation, and matched the patterns of enriched cellulosic stable carbon isotope indicative of physiological stress. While winter and summer temperature and summer precipitation influenced pathogen dynamics at all sites, the primary climatic factor of these three limiting factors varied spatially by location, topography, and elevation. In the 20^th^ century, SNC impacts at low- to mid-elevations were least severe during the warm phase of the Pacific Decadal Oscillation (PDO, 1924–1945) and most severe in 1984–1986, following the cool phase of the PDO (1945–1977). At high elevations on the west slope of the Cascade Mountains, SNC impacts were the greatest in the 1990s and 2000s, a period of warmer winter temperatures associated with climate change. Warmer winters will likely continue to increase SNC severity at higher elevations, north along the coast from northern Oregon to British Columbia, and inland where low winter temperatures currently limit growth of the pathogen. Surprisingly, tree-ring records of ancient Douglas-fir logs dated ~53K radioactive years B.P. from Eddyville, OR displayed 7.5- and 20-year periodicities of low growth, similar to those found in modern day coastal Douglas-fir tree-ring records which we interpret as being due to cyclic fluctuations in SNC severity. Our findings indicate that SNC has persisted for as long as its host, and as a result of changing climate, may become a significant forest health problem in areas of the PNW beyond the coastal fog zone.

## Introduction

Swiss needle cast (SNC) is an economically important disease of most forms of Douglas-fir (*Pseudotsuga menziesii* (Mirb.) Franco, *P. menziesii* var. *glauca* [Beissn.] Franco, and *Pseudotsuga macrocarpa (Vasey) Mayr*) [1,2]. SNC is caused by the fungus *Nothophaeocryptopus gaeumannii* (Rhode) Petrak and occurs wherever its host is found, but historically has been of minor importance in western North American forests [1,3]. Epidemic outbreaks of SNC have been reported in coastal Oregon, Washington, and British Columbia and have steadily increased in severity since ~1984 [3–6]. Disease is most severe in forests and plantations on the western slopes of the Oregon Coast range within the coastal fog zone [3]. The affected area with visible SNC symptoms - chlorosis and premature needle loss - seen from annual aerial surveys of coastal Oregon have set new record highs each of the last six years [7]. Evidence suggests that SNC is affected by climate [8,9], alone or in combination with forestry practices of the (later) 20^th^ century. Possible causes for the current increase in SNC severity include: climate warming and the introduction of Christmas tree plantations in the mid-1970s interacting with high soil nitrogen [10,11], or genetic changes in the pathogen [12]. There is mounting concern that SNC is increasing in severity, frequency and range in association with rising winter temperatures (and spring precipitation) and will continue to intensify over the 21^st^ century due to climate change [13,14].

While the epidemiology of SNC and mechanisms of pathogenicity of *N. gaeumannii* on Douglas-fir have been well studied in young plantations, knowledge remains limited on the history and spatial distribution of SNC impacts on mature and older tree growth in the Pacific Northwest (PNW). *N. gaeumannii* is indigenous in western North America and has long believed to have been pervasive but innocuous in Douglas-fir forests prior to 1950 [1,15,16]. Increased severity since ~1950 is thought to be at least in part, climate mediated because the causal fungus is sensitive to small differences in temperature and moisture [5,8,9]. A recent dendrochronological study indicates that SNC has affected Douglas-fir growth at least back to 1592, which was the earliest of the available tree-ring records [17]. Furthermore, the spatiotemporal patterns of decreased annual ringwidth associated with SNC are synchronous across six coastal sites in Oregon representing a latitudinal transect at varying elevations, display periodicities of 25–30 years, and are strongly correlated with winter and summer temperatures and summer precipitation. SNC impacts as measured by tree ring width in coastal Oregon peaked in 1984–1986-thought to be a period when the fungal population reached epidemic levels following several decades of environmental conditions favorable to growth and reproduction of *N. gaeumannii*[17].

Growth reduction of Douglas-fir due to SNC in the PNW is symptomatic in the Coast Range of Oregon and Washington, primarily, and is of limited concern outside this region [6]. There has been no reported evidence of SNC impacts on growth of inland Douglas-fir although symptoms are often noted in plantations in Northern Idaho and Western Montana [18]. A broad scale study involving 59 young Douglas-fir stands (10–23 years) found no growth reductions in the Oregon Cascades during a SNC outbreak between 2001 and 2006 [19].

Here, we extend the dendrochronological findings of Lee, et al. [17] to examine the history and spatial extent of the cyclical pattern of SNC outbreaks in association with the seasonal climate factors and address the three questions in the panel titled, “Where? Why there? Why now?”,” We conducted a dendrochronological study to test the following hypotheses: (1) *N. gaeumannii* is an ancient tree pathogen that affects Douglas-fir growth as far back as its earliest known existence in the Paci ic Northwest; (2) SNC is ubiquitous and affects Douglas-fir growth across western Oregon from the Coast Range to the Cascade Range; (3) SNC is sensitive to winter and summer temperature, and summer precipitation, and so, spatial variability in SNC severity can be attributed to variations in site conditions and location. We developed new master chronologies of ancient and modern Douglas-fir ringwidth from the west slopes of the Coast Range to the west slopes of the Cascade Range of Oregon. We examined the spatial distribution of SNC impacts on mature Douglas-fir trees using time series intervention analysis of intra-annual tree ringwidth chronologies to reconstruct the history of SNC impacts by site.

### The disease cycle

**Figure 1:**
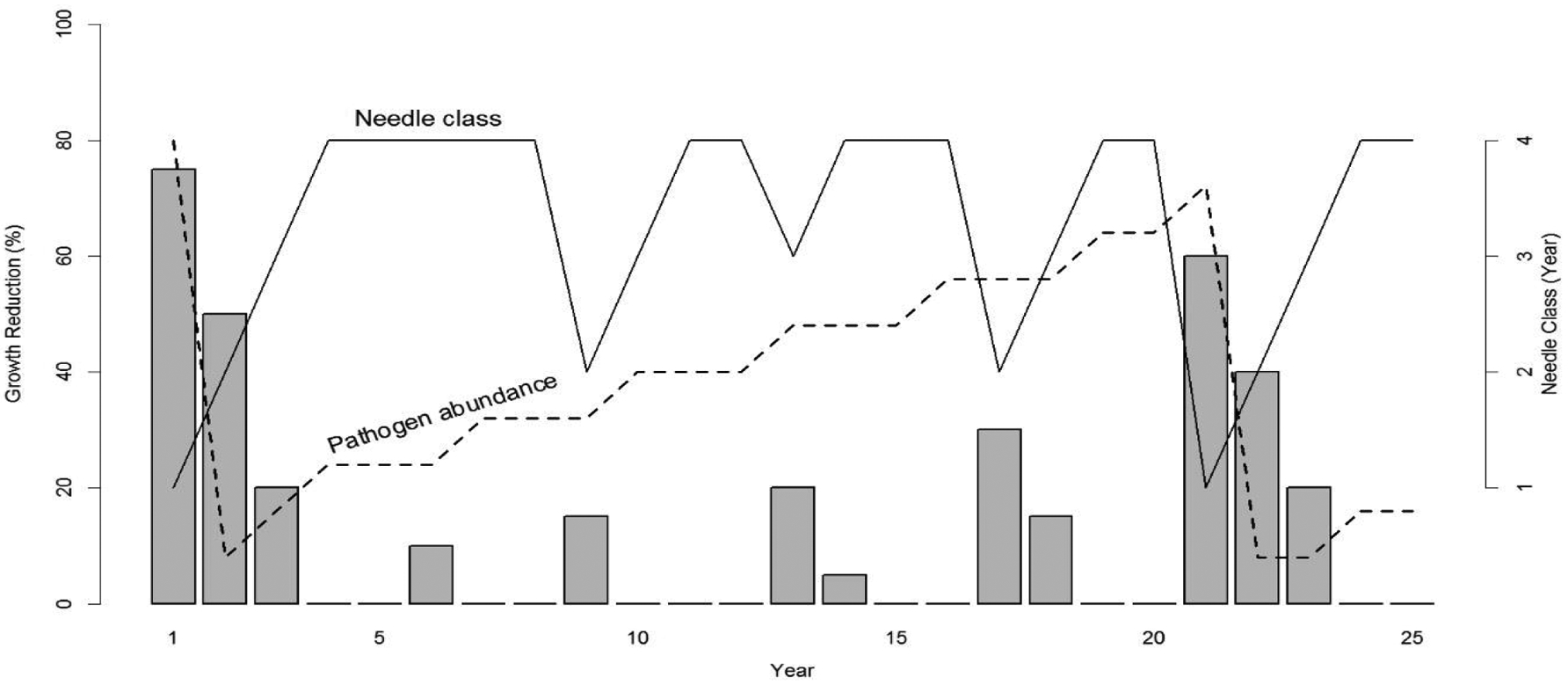
Conceptual model of Swiss needle cast (SNC) impact on tree growth in association with the abundance of *Nothophaeocryptopus gaeumannii* and number of needle classes retained [17]. The number of needle classes retained varies from one (when the tree is heavily infected) to four (least infected). Pathogen abundance increases from endemic (when two-year-old and older needles are abscised) to epidemic levels (when tree is heavily infected) over several decades. The disease cycle begins anew with a peak reduction in growth when pathogen abundance reaches epidemic levels and is then reset to endemic levels following the early abscission of two-year-old and older needles. Growth reductions display 4- and ~20-year periodicities because *N. gaeumannii* infects only the newly emerged needles at time of sporulation and has a four-year life cycle.

The key growth pattern in tree-ring records of coastal Douglas-fir is a sinusoidal cycle of anomalously low growth having a primary periodicity of ~25–30 years and a harmonic periodicity of ~4 years associated with SNC [17]. The cyclical patterns of SNC impact on Douglas-fir growth occur throughout the life of the tree and because of the effects synoptic seasonal weather patterns on fungal growth, are synchronous across coastal Oregon. We combined our dendrochronological indings with the epidemiology of SNC to develop a conceptual model of the disease cycle driven by needle retention and fungal fruiting body abundance which have routinely been used as indices of disease severity [3,8,11,16]. SNC reduces assimilation of carbon and tree diameter by stomatal occlusion and early needle abscission [3,8]. Consequently, yearly changes in SNC impacts depend upon inoculum abundance, ascospore germination, and pathogen colonization in association with climatic conditions which affect the proportion of stomata occluded and needle retention. Douglas-fir trees on the coast typically retain up to four years of needles but may only have current and 1-year-old foliage due to premature needle abscission in severely affected plantations [3,13,20]. In our conceptual model, the disease cycle begins when pathogen abundance is at epidemic levels, resulting in loss of 2-year-old and older needles and a significant reduction in stem growth (Figure 1). The pathogen population will be reduced due to premature needle abscission resulting in fewer infected needles and a reduction in inoculum. Peak SNC outbreaks reduce tree growth for several consecutive years because photosynthetic capacity is restored to normal only after all needle classes have formed [21]. A delay of several years between inoculation and growth of the fungus and tree growth reduction is expected because the pathogen infects only the newly emerged needles [9,22]. This lagged growth response to SNC is represented by a 4-year periodicity in disease impacts (Figure 1). The slow buildup of pathogen abundance from endemic to epidemic levels over several generations is represented by a 20-year periodicity. The dominant periodicity of 20 years varies by site and is as low as 6 years at Tillamook where more favorable climatic conditions allow the fungus to develop faster [9,17]. Pseudothecia can be commonly found on 4 to 7-year-old needles in the Cascade Range of Oregon and Washington, and on 1 to 2-year-old needles in some areas of the Coast Range where pathogen dynamics are enhanced by more favorable climatic conditions [9]. Pathogen abundance is not reset to endemic levels by abscission of 2-year-old and older needles in areas where disease is constantly severe as indicated by a < 10-year disease cycle and the presence of pseudothecia on 1 to 2-year-old needles.

### Epidemiology of SNC and climate relations

**Figure 2:**
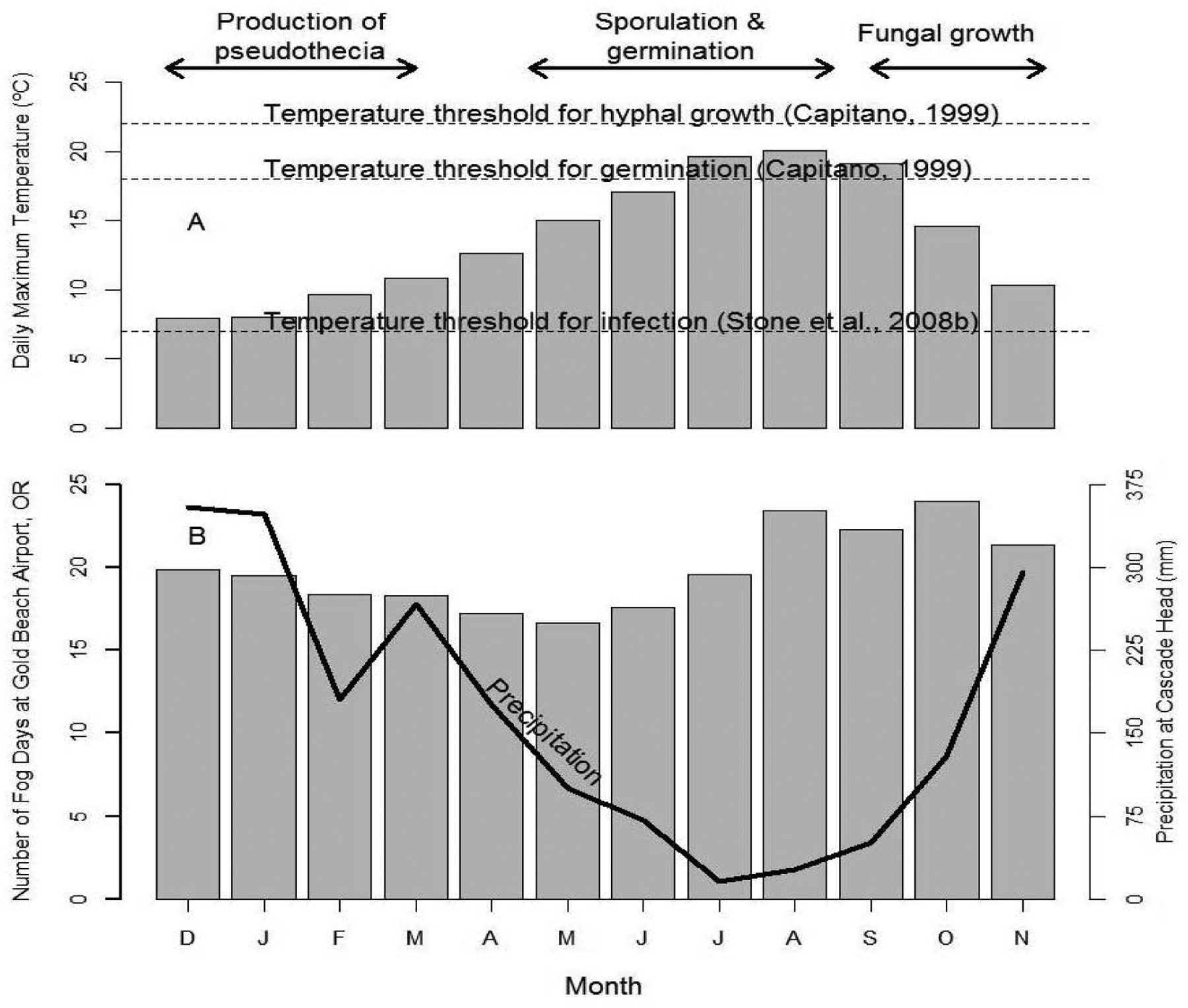
Seasonal pattern of (A) temperature, (B) precipitation and fog frequency at Cascade Head on west slopes of Coast Range of Oregon in relation to the three developmental stages of *Nothophaeocryptopus gaeumannii*. The climatic factors limiting pathogen dynamics are winter (November-February) and summer (June-July) temperatures and summer (June-July, primarily July) needle wetness.

**Figure 3:**
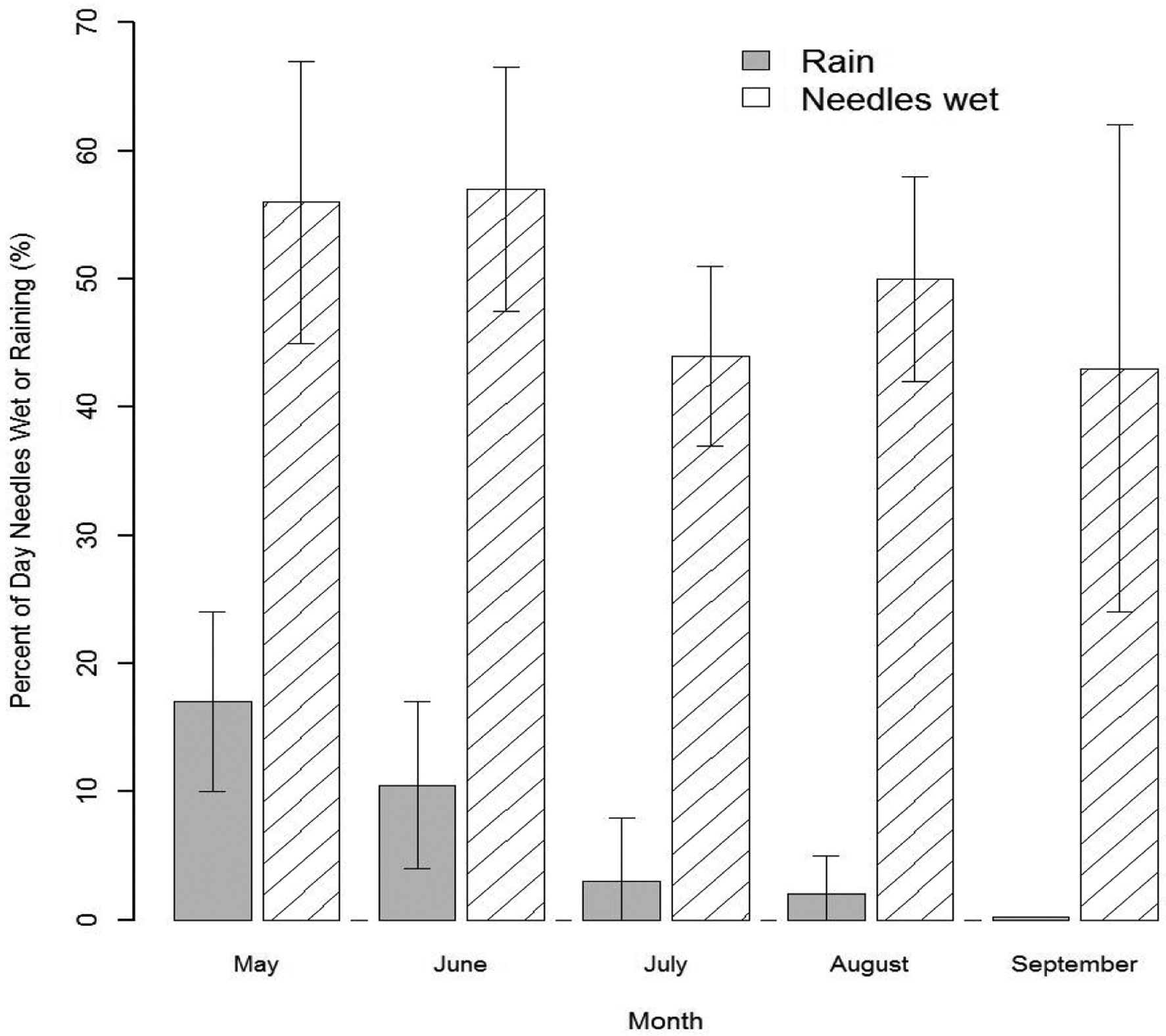
Percent of day when precipitation occurs versus when needles are wet in the summer of 2014 at Cascade Head. Needle wetness is maintained by coastal fog during the annual summer drought period.

Three major phases of the infection cycle of *N. gaeumannii* are relevant to the climate-growth relation [8]: (1) the fungus reproduces only sexually and pseudothecia (i.e., fruiting bodies) develop in winter and can begin plugging stomata as early as December; (2) sporulation and initial infection of needles occur from May to July; and (3) needle colonization by internal hyphal growth occurs year round following initial infection (Figure 2). Wet needles in late spring and early summer are necessary for spore dispersal and initial infection via the stomata [9,23]. Mild winters, spring precipitation, and moderate summer temperatures at a coastal site on west slopes of the Coast Range are highly favorable conditions for *N. gaeumannii* (Figure 2A). While precipitation is steadily decreasing during sporulation in the summer, fog frequency is steadily increasing (Figure 2B). Needle wetness is maintained by coastal fog in late summer and is less a limiting factor of fungal development along the coast (Figure 3). SNC impacts on Douglas-fir growth are most severe along the coast where winter daily maximum temperatures are above 7 °C [9], summer temperatures range between the temperature optima for germination (18 °C) and growth (22 °C) [23], and summer needle wetness is adequate for fungal colonization of needles.

### Reconstruction of SNC impacts on Douglas-fir growth in coastal oregon

**Figure 4:**
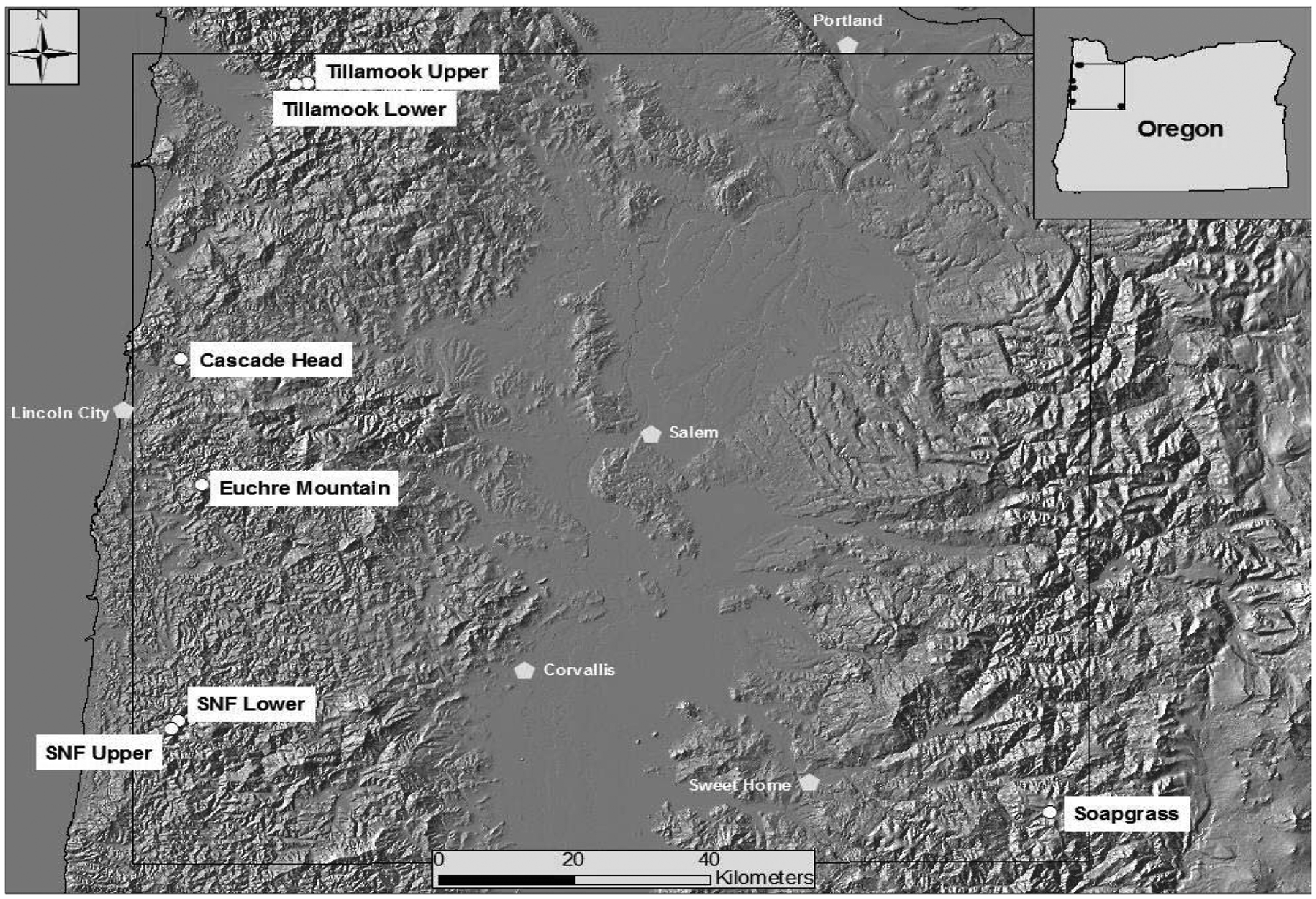
Locations of six study sites in Oregon Coast Range [17]. One reference Douglas-fir site, Soapgrass Mountain, is located at about 1200 m elevation on the western slope of the Cascade Range of Oregon.

**Figure 5:**
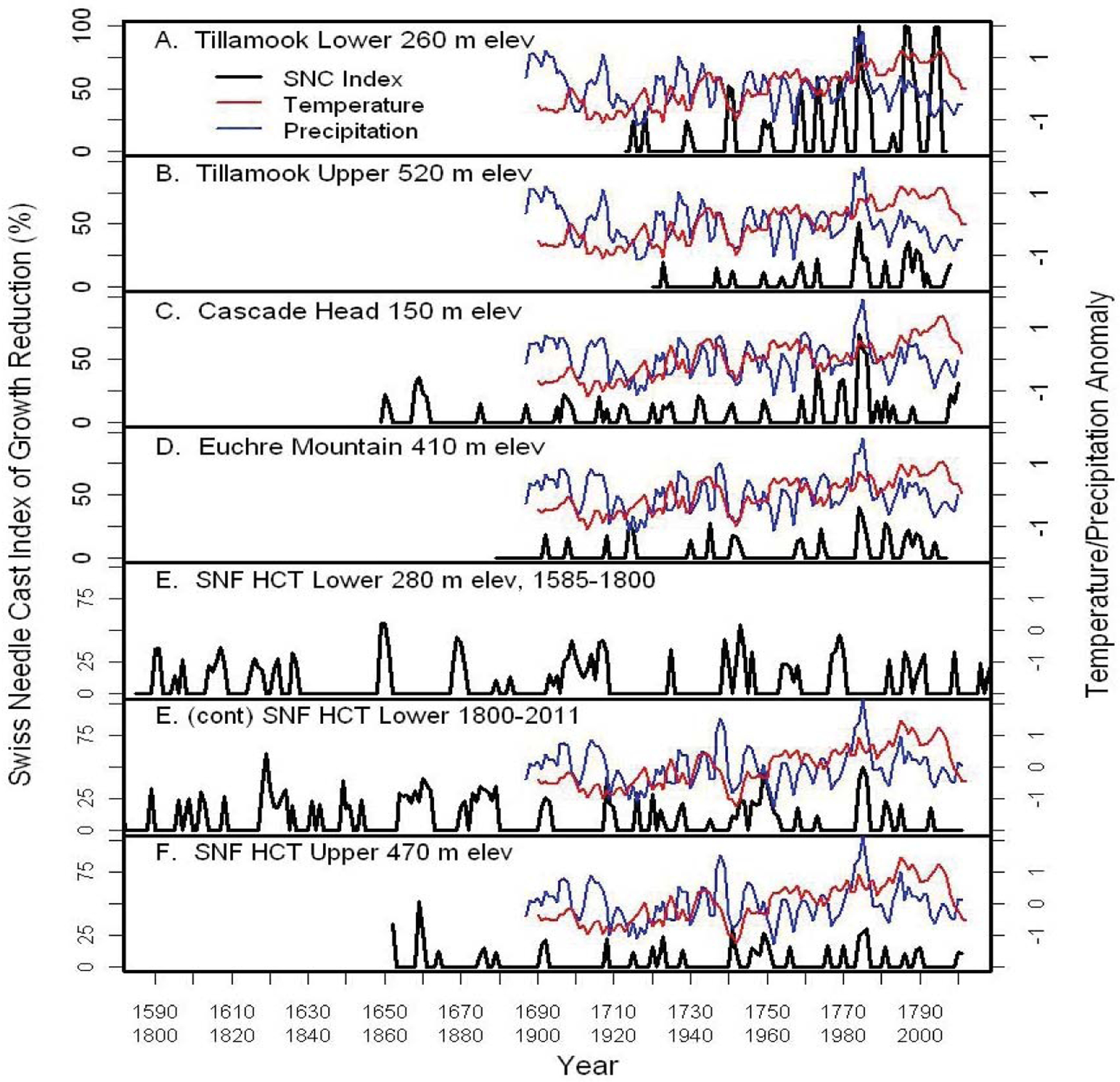
The percent reduction in annual tree-ring width increment of Douglas-fir adjusted for temperature and a climate proxy at six study sites in coastal Oregon [17]. Temperature and precipitation were normalized to a mean of 0 and a variance of 1. The red line is the 5-year running average of mean daily maximum temperature for January and February. The blue line is the 3-year running average of total precipitation for June and July.

We analyzed tree-ring chronologies from six late-successional Douglas-fir stands in the western Oregon Coast Range using Time Series Intervention Analysis (TSIA) to address how climate relates to the impact of SNC on tree growth (Figure 4) [17]. Tree-ring chronologies of western hemlock (*Tsuga heterophylla*), a species not susceptible to the fungus *Nothophaeocryptopus gaeumannii*, and Douglas-fir at Soapgrass Mountain, a high Cascades site, were used as a climate proxy in the TSIA. We found that growth reductions associated with SNC dated back to the 1590s, the earliest record in our dendrochronological data (Figure 5). Growth reductions were synchronous across the six sites indicating that the disease severity was influenced by regional climatic conditions. SNC impact peaked in 1984–1986 at all six study sites, followed by unprecedented disease impacts of 100% in 1996 and 2004 at one site, while decreasing to previous levels at the other five sites. SNC impacts displayed cyclical patterns having periodicities of 6, 12, and 25–30 years which were coherent across the region and represented the disease cycle unique to SNC (Figure 5). The synchronization of SNC impact on Douglas-fir across the landscape indicated that there were climate factors, which favored disease conditions at these sites in coastal Oregon.

### SNC impacts on ancient Douglas-fir

**Figure 6:**
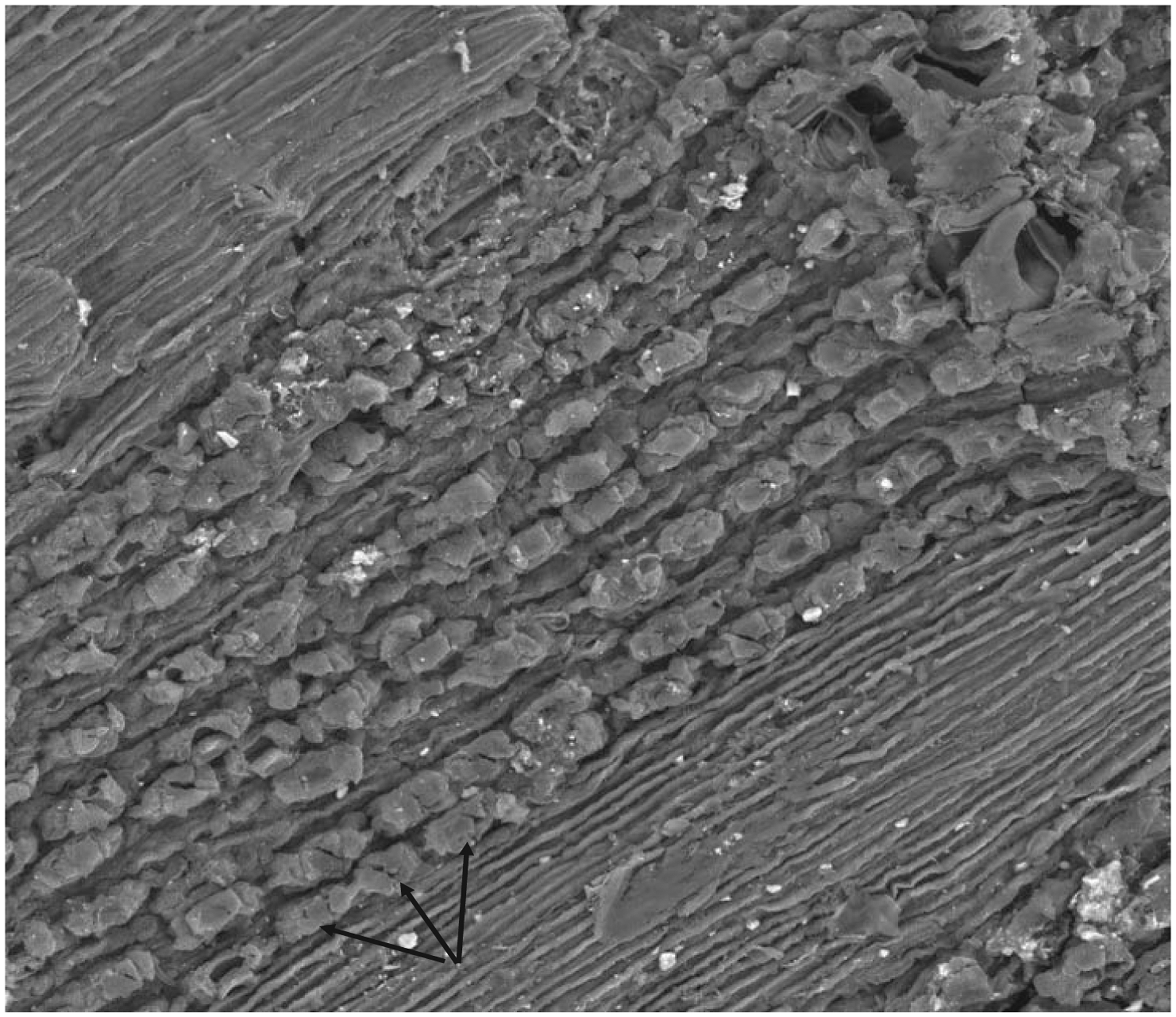
Scanning electron micrograph of pseudothecia primordia (see arrows) blocking the stomata of an ancient Douglas-fir needle found 29 m below the surface in landslide deposits near an Oregon Department of Transportation highway reconstruction project by Eddyville, Oregon. Image courtesy of William Rugh.

**Figure 7:**
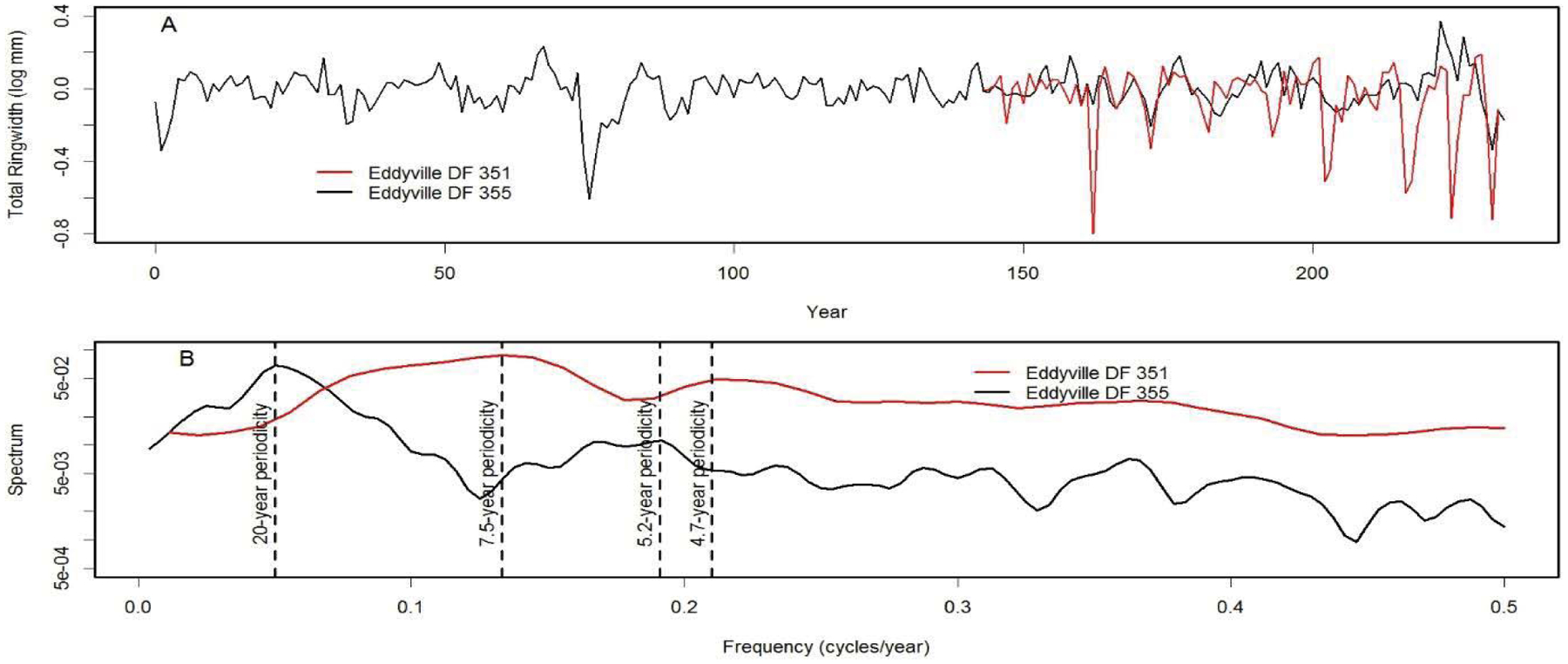
(A) Master chronologies of two ancient Douglas-fir trees from Eddyville, Oregon and (B) comparison of their spectrum. The chronology of tree 351 diverges from that of tree 355 and displays a cyclical pattern of anomalously low growth having a primary periodicity of 7.5 years and secondary periodicity of 4.7 years. Tree 355 displays a cyclical pattern having a primary periodicity of 20 years and a secondary periodicity of 5.2 years.

**Figure 8:**
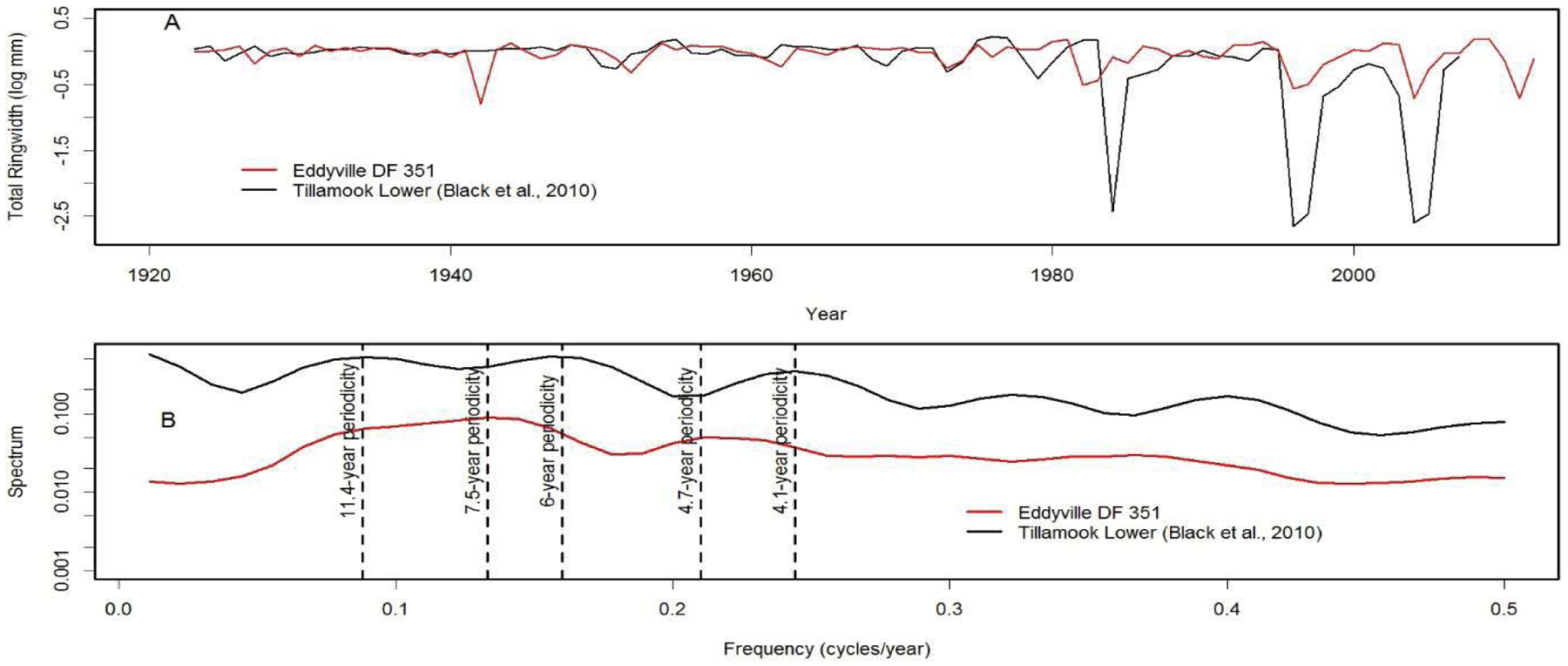
(A) The modern day analog of the ancient Douglas-fir tree 351 from Eddyville, Oregon is the master chronology of Douglas-fir at Tillamook Lower which displays cyclical patterns of anomalously low growth attributed to Swiss Needle Cast [5]. (B) The disease cycles at Tillamook and Eddyville have a similar primary periodicity of 6 and 7.5 years, respectively, and a secondary periodicity of 4.1 and 4.7 years, respectively. The disease cycle at Tillamook has another primary periodicity of 11.4 years which is less pronounced than at Eddyville.

We analyzed tree-ring chronologies from two of 11 Douglas-fir logs that were unearthed in 2008–2010 by the Oregon Department of Transportation along the U.S. Highway 20 reconstruction site due east of Eddyville, OR (N44°39ʹ, W123°47ʹ). The logs, needles, and seed cones were encased in ancient landslide deposits at 26 m below the surface and were remarkably preserved. Radiocarbon dating estimates ages ~53K Before Present (BP) in the Marine Isotope Stage 3 (MIS3, ca. 60 to 27 K BP) period which was generally cold but with intermittent Dansgaard-Oeschger warm phases [24]. Stem diameters range from 64 cm for the 89 year-old log (351) to 128 cm for the 233 year-old log (355) and are comparable in size to contemporary coastal Douglas-fir trees of the same age. The similar growth rates indicate that the ancient Douglas-fir come from a temperate rainforest environment comparable to present day. Needles of the ancient Douglas-fir appear to have significant stomatal occlusion by structures resembling pseudothecia of *N. gaeumannii*, as seen under a scanning electron microscope (Figure 6). The two tree-ring series were successfully cross-dated but diverged and displayed periodicities of either 7.5 or 20 years (Figure 7), indicative of a non-climatic forest disturbance agent that affected one tree (351) more than the other (355). The chronology of tree 351 displays several 3-year periods of growth reduction approximately every 7.5 years. The 7.5-year disturbance cycle is similar to that of Tillamook Lower which has a periodicity of 6 years caused by SNC (Figure 8). The 20-year disturbance cycle of tree 355 is similar to the 25-year SNC cycle at Horse Creek Trail Lower in the Siuslaw National Forest (not shown). We attribute the 7.5 and 20 year disturbance cycles of the ancient Douglas-fir to SNC because the periodicities of low growth are similar to those of the SNC disease cycles of contemporary Douglas-fir and the stomata are possibly occluded. Furthermore, the anomalously low growth years of tree 351 are not synchronous with those of tree 355, indicating a non-climatic stress on individual trees rather than a climatic stress on all trees.

### SNC impacts Douglas-fir inland

**Figure 9:**
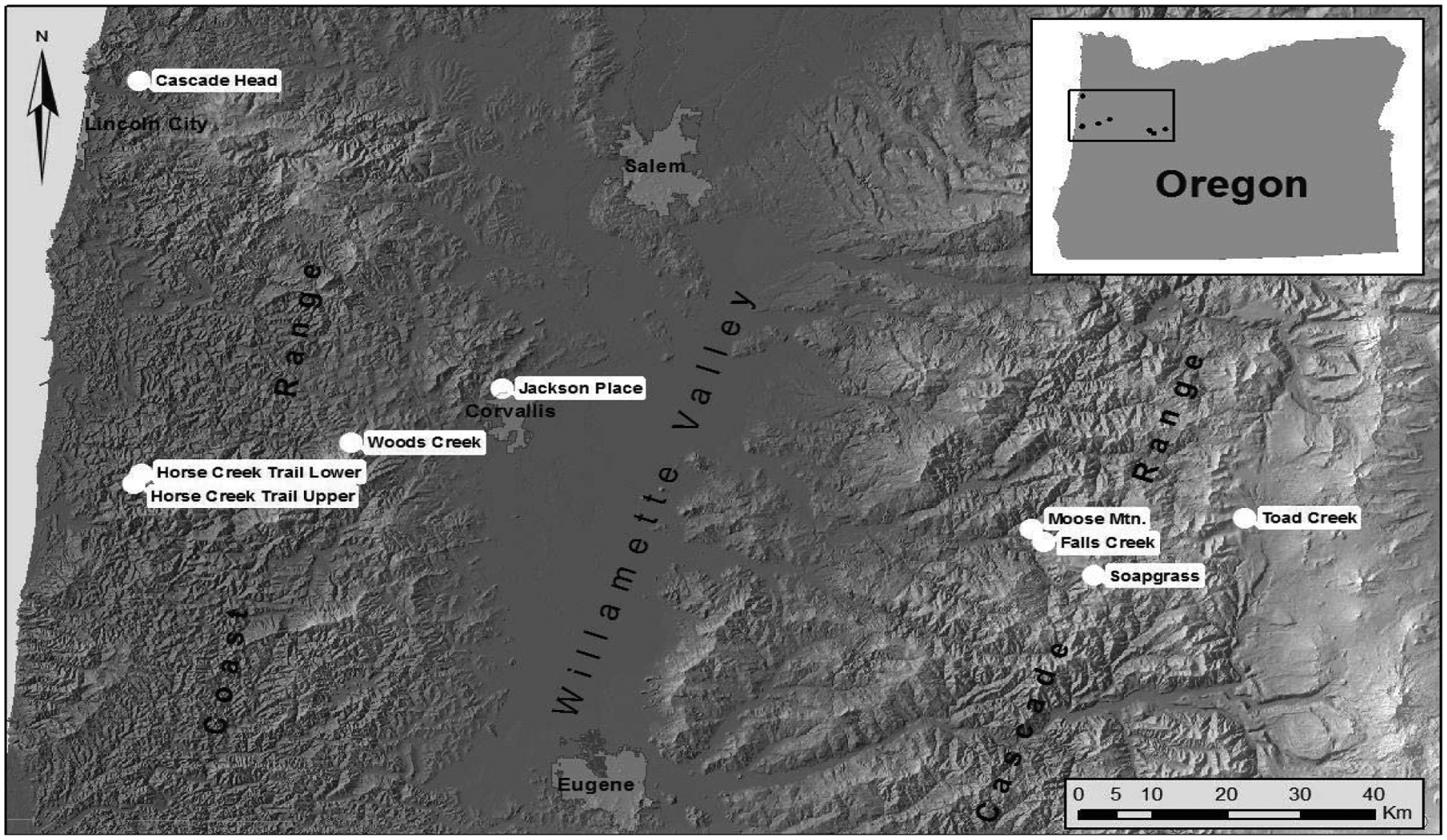
Tree core samples were collected from nine field sites located in mature Douglas-fir stands on the west and east sides of the Coast Range, in the Willamette Valley, and on the west side of the Cascade Mountains.

We examined the spatial distribution of SNC impacts on mature Douglas-fir trees using TSIA of earlywood (EW) and latewood (LW) ringwidth chronologies from the west slope of the Coast Range to mid- and high-elevations on the west slope of the Cascade Mountains of Oregon (Figure 9). The EW and LW series represent a seasonal time series with a mean response function that contains components for climate and SNC outbreaks. The spatially-explicit predicted growth response to temperature and water was used as a climate proxy and was subtracted from the master chronology to isolate the disease signal. All sampled stands experienced signi icant radial growth reductions in Douglas-fir that could not be accounted for by current and previous-year seasonal climatic factors. The spatiotemporal patterns of growth reduction attributable to SNC were synchronous across the region, displayed periodicities of 25–30 years, and were strongly correlated with winter and summer temperatures and summer precipitation. Our indings indicate that detectable SNC impacts occur wherever Douglas-fir is found in western Oregon and is not limited to the coastal fog zone.

### Climate relations with SNC

**Figure 10:**
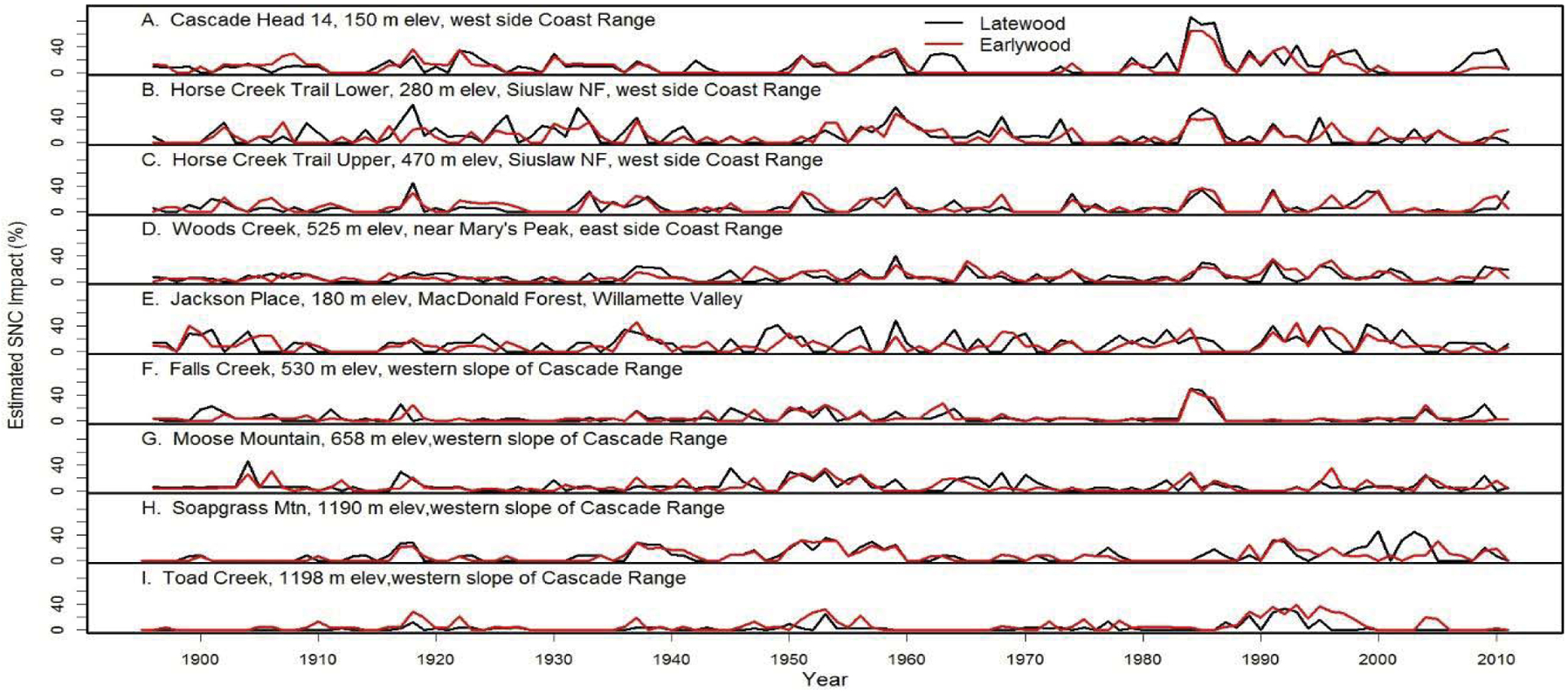
The percent reduction in earlywood and latewood ringwidth increment of Douglas-fir attributed to Swiss Needle Cast at nine study sites from the west slopes of the Coast Range to the west slopes of the Cascade Mountains of Oregon. The growth anomalies could not be explained by seasonal climate variables for temperature and water. Peak SNC impacts occurred in 1918, 1959, and 1984–1986 approximately 25–41 years apart and were synchronous across the region.

To infer the climate relations with SNC, we used TSIA to classify each year into one of three disease states, SNC growth suppression, no suppression, and release. The dominant pattern of Douglas-fir growth at each site was a disease cycle with a primary periodicity of 25–30 years and secondary periodicity of ~4 years (Figures 5,7,8,10) that is the interaction of climatic and non-climatic factors (Figure 1). We hypothesize that changes in pathogen abundance, amount of inoculum, needle class retention, stomatal occlusion, and climate over one or more decades cause the cycling of disease states throughout the life of the tree. To determine the climate relations with SNC, it was necessary to isolate the climate effects which were confounded with the biotic effects. The years classified as no SNC suppression or release have a SNC index value of 0% and do not correlate with climate. Consequently, the climate relations with SNC were determined using only the years classified as SNC suppression which have a negative pulse intervention resulting in a positive SNC index value. According to the conceptual disease cycle model, growth response to SNC was lagged and was the culmination of the interaction of climatic and biotic factors over multiple decades. Rather than correlating the nonzero SNC index with seasonal temperature and precipitation in the current and each previous year, we calculated the canonical correlations of SNC index with temperature and precipitation for the current and previous 30 years by site [17].

**Figure 11:**
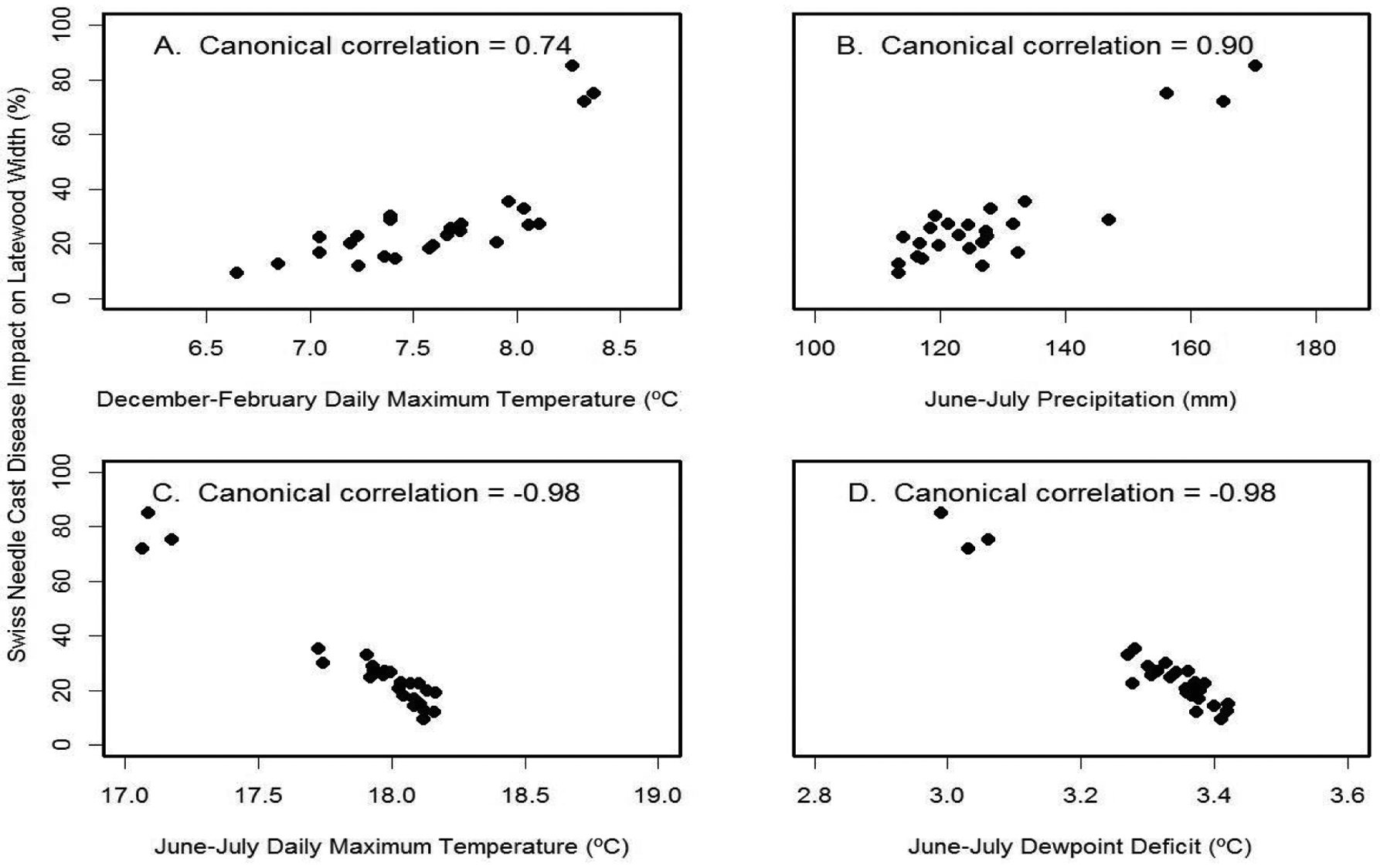
Canonical correlation of SNC index of impact on latewood growth with (A) winter temperature, (B) summer precipitation, (C) summer temperature, and (D) summer dewpoint deficit at Cascade Head. Temperature and precipitation were summarized on a seasonal basis so as to maximize the canonical correlations with the SNC index.

For Cascade Head, the SNC index of impact on latewood growth correlated best with the canonical variables for June-July temperature (r_can_=−0.98) and June-July dewpoint de icit (r_can_=−0.98) (Figure 11). The multiple regression equation of SNC impact on climate was

SNC index=815−47.1 JJ_Temp+4.3 DJF_Temp+0.19*JJ_Prec

and accounted for 96% of the variation where JJ_Temp= June-July mean daily maximum air temperature (°C), DJF Temp= December-February mean daily maximum air temperature (°C), and JJ_Prec= June-July total precipitation (mm). The key explanatory variable was June-July temperature based on Kruskal’s measure of relative importance, indicating that high summer temperatures reduce the SNC impacts on Douglas-fir growth at this coastal site. While summer temperature and precipitation were correlated, identification of the key climatic factors associated with SNC was possible because the long history of SNC impacts represented a century that had high climatic variability as well as high variability in the tree-ring records.

**Figure 12:**
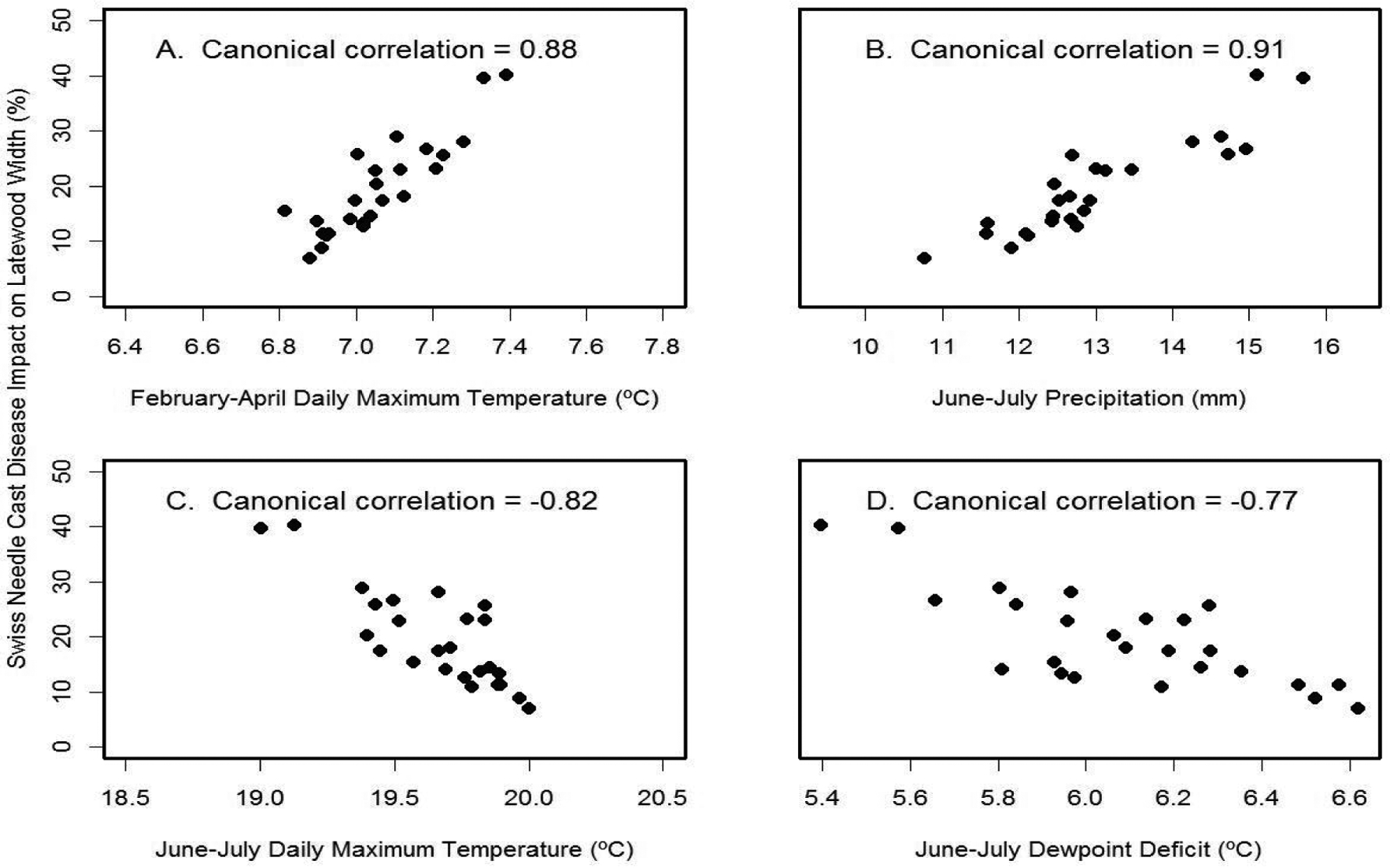
Canonical correlation of SNC index of impact on latewood growth with (A) winter temperature, (B) summer precipitation, (C) summer temperature, and (D) summer dewpoint deficit at Soapgrass Mountain. Temperature and precipitation were summarized on a seasonal basis so as to maximize the canonical correlations with the SNC index.

For the high Cascades site, Soapgrass Mountain, the SNC index correlated best with the canonical variables for June-July precipitation (r_can_=−0.91) and February-April temperature (r_can_=0.88) (Figure 12). The multiple regression equation of SNC impact on climate was

SNC index=−30−9.2 JJ_Temp+26 FMA_Temp+2.5*JJ_Prec

and accounted for 95% of the variation where JJ_Temp= June-July mean daily maximum air temperature (°C), FMA_Temp= February-April mean daily maximum air temperature (°C), and JJ_Prec= June-July total precipitation (mm). The key explanatory variable was February-April temperature based on Kruskal’s measure of relative importance. The multiple regression results differed from the canonical correlation results because correlation considers one factor at a time whereas regression considers all factors simultaneously. Note also that the winter months of importance at Soapgrass were different than those at Cascade Head because temperatures in the two coldest months, December and January, were below the growth threshold in the high Cascades. Consequently, pseudothecia of *N. gaeumannii* likely formed one to two months later due to the colder environment.

**Figure 13:**
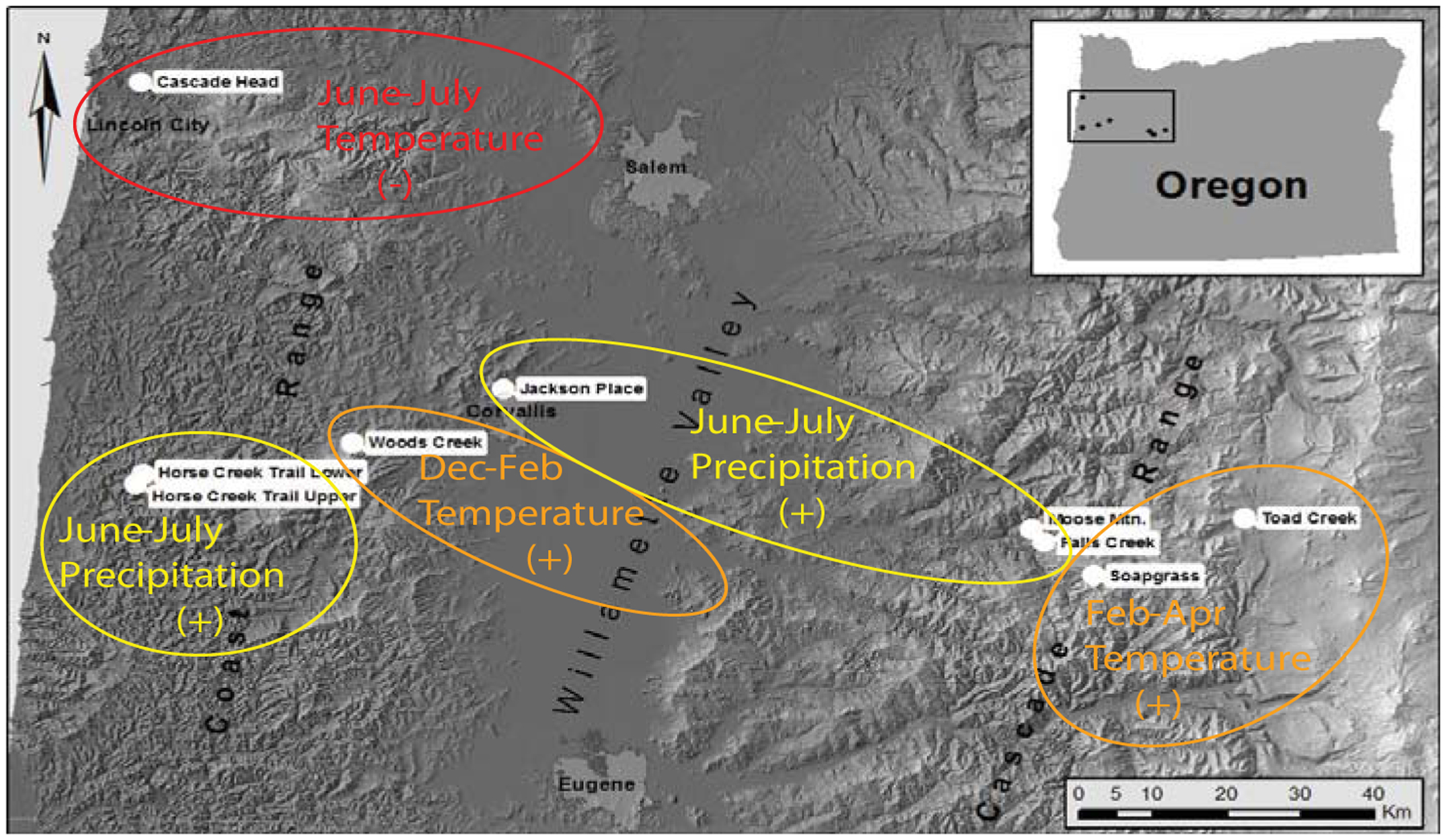
Map of study sites indicating the primary climatic factor that is most limiting to fungal development at each site based on time series intervention analysis of dendrochronological data.

Winter temperature, summer temperature and precipitation are the key limiting factors of pathogen abundance at all sites but, of these three climate factors, the primary limiting factor varies by site conditions and location (Figure 13). Summer precipitation is most limiting in warm, dry environments in the Willamette Valley and in some coastal sites in southern Oregon where summer needle wetness is less maintained by coastal fog. Winter temperature is most limiting in cool environments on the east slopes of the Coast Range and above the snowline on the west slopes of the Cascade Mountains. Summer temperature is most limiting at one coast site that lies more within the coastal fog zone.

### SNC is influenced by the Pacific Decadal Oscillation (PDO)

**Figure 14:**
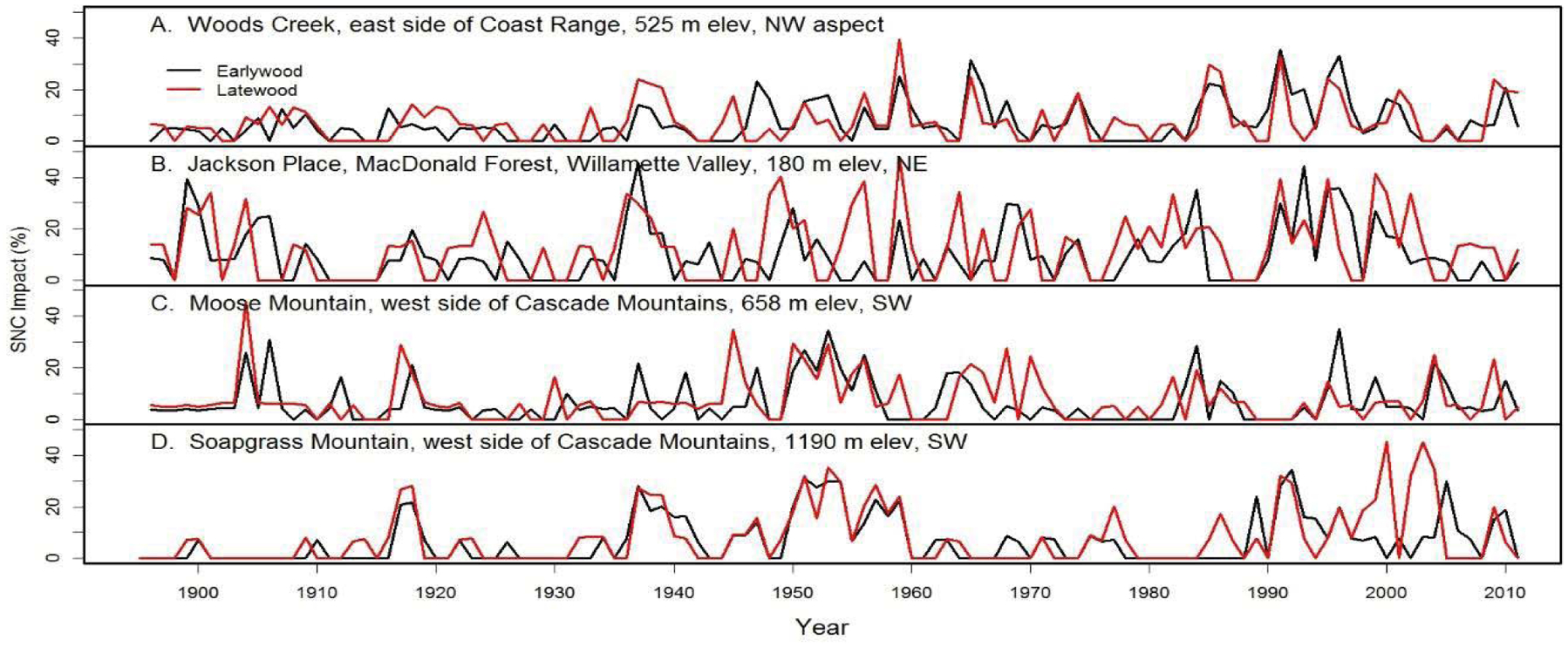
Swiss needle cast (SNC) index of impact at four inland sites display disease cycles having a 25–30 year periodicity, a relationship with the Pacific Decadal Oscillation (PDO), as well as an increasing trend at two sites, Woods Creek and Soapgrass Mountain. SNC impacts were less frequent and severe between 1925 and 1946 during a strong warm phase of the PDO than between 1947 and 1978 during a strong cold PDO phase.

In the 20^th^ century, the PNW has experienced high climatic variability including a strong warm phase of the PDO (1925–1946), followed by a strong cool phase (1947–1978). We found an inverse relation between PDO and SNC effects on tree-ring width, i.e., reduced effects during the warm phase and increased effects during the cool phase (Figure 14).

The period from 1917 to 1940 was exceptionally warm and dry and the drought of the 1930s was the second most severe drought of the last 250 years [25], likely resulting in less SNC impact on tree growth (Figure 14). This was followed by several wet periods from 1941 to 1955 and from 1968 to 1984, likely resulting in greater SNC impact and culminating with the 30-year peak impact in 1984–1986. The linkage between cool PDO phases and increased SNC impact continued after 1984 as evidenced by the intensi ication of SNC impacts on the east side of the Coast Range and in the high Cascades in recent decades during a mostly cool PDO phase (1998–2014) (Figure 14). However, the positive trend in SNC impacts at two sites (Woods Creek, Soapgrass Mountain) was more influenced by increasing winter temperatures due to climate warming than by summer conditions which were more favorable to fungal develop in part due to the cool PDO phase.

## Conclusion

SNC impacts occur wherever Douglas-fir is found and are synchronous across western Oregon, indicating that SNC is in luenced by regional climate. SNC impacts in the PNW date back to ~53K radioactive years BP as evidenced by the cyclical patterns of low growth in the master chronologies of ancient Douglas-fir that match the modern SNC disease cycles at coastal sites, and supported by the presence in the ancient needles of putative pseudothecia of *N. gaeumannii*. This long history of SNC predates forest management practices and improves our understanding of the climate factors affecting the causal fungus. SNC impacts on Douglas-fir growth as seen in tree-rings display 6 to 30 year periodicities throughout the life of the tree. The higher frequencies in the disease cycle represent a lagged growth response to SNC caused by the infection of only the newly emerged needles at time of sporulation, followed by colonization of the needle over several years which is unique to *N. gaeumannii*. With warmer winters, SNC impacts are increasing in mature closed-canopy Douglas-fir stands on the east slopes of the Coast Range and in the high Cascades. Temperatures will likely continue to increase due to climate change, and consequently, SNC is expected to intensify in frequency and magnitude at higher elevations, north along the coast from northern Oregon to British Columbia, and inland where current winter temperatures limit fungal growth.
